# Clinical utility of DNA methylation profiling for choroid plexus tumors

**DOI:** 10.1093/noajnl/vdae097

**Published:** 2024-06-12

**Authors:** Kee Kiat Yeo, Cassie B Macrae, Bradley Gampel, Jared T Ahrendsen, Hart Lidov, Karen D Wright, Susan Chi, Katie Fehnel, Lissa Baird, Jessica Clymer, Kenneth Aldape, Sanda Alexandrescu

**Affiliations:** Department of Pediatric Oncology, Dana-Farber/Boston Children’s Cancer and Blood Disorder Center, Boston, Massachusetts, USA; Department of Pathology, Boston Children’s Hospital, Boston, Massachusetts, USA; Department of Forensic Pathology, University of New Mexico, Albuquerque, New Mexico, USA; Department of Pediatric Oncology, Dana-Farber/Boston Children’s Cancer and Blood Disorder Center, Boston, Massachusetts, USA; Department of Pediatrics, Sylvester Comprehensive Cancer Center, Miami, Florida, USA; Department of Pathology, Northwestern University Feinberg School of Medicine, Chicago, Illinois, USA; Department of Pathology, Boston Children’s Hospital, Boston, Massachusetts, USA; Department of Pediatric Oncology, Dana-Farber/Boston Children’s Cancer and Blood Disorder Center, Boston, Massachusetts, USA; Department of Pediatric Oncology, Dana-Farber/Boston Children’s Cancer and Blood Disorder Center, Boston, Massachusetts, USA; Department of Neurosurgery, Boston Children’s Hospital, Boston, Massachusetts, USA; Department of Neurosurgery, Boston Children’s Hospital, Boston, Massachusetts, USA; Department of Pediatric Oncology, Dana-Farber/Boston Children’s Cancer and Blood Disorder Center, Boston, Massachusetts, USA; Department of Pediatrics, New York University Lagone Medical Center, New York, New York, USA; Center for Cancer Research, National Cancer Institute, Bethesda, Maryland, USA; Department of Pathology, Boston Children’s Hospital, Boston, Massachusetts, USA

**Keywords:** choroid plexus neoplasms, clinical outcome, DNA methylation, TP53

## Abstract

**Background:**

Choroid plexus tumors (CPTs) are rare, potentially aggressive CNS tumors with defined histologic criteria for grading. In recent years, several patients within our practice have demonstrated discordance between the histologic diagnosis and clinical behavior. DNA methylation profiling has emerged as a potential diagnostic adjunct for aiding the clinical approach.

**Methods:**

We reviewed the clinical and pathologic data of all CPTs diagnosed at Boston Children’s Hospital from 1995 to 2023. All cases with available material (38/48) underwent DNA methylation profiling at NIH/NCI, and the classifier results were correlated with the WHO histologic grade and patient outcomes. Survival information was analyzed using Kaplan–Meier curves.

**Results:**

There was good correlation (11/12, 92%) between methylation class and WHO histologic grade for choroid plexus carcinomas (CPC); one histologic CPC grouped with choroid plexus papilloma (CPP) group pediatric (P). Five CPPs grouped with methylation class CPC (5/17, 29%). In the group of atypical CPPs (*n* = 9), there were two that grouped with methylation class CPC. Survival analysis showed utility of methylation classes in the prediction of biologic behavior.

**Conclusions:**

Results indicated that methylation profiling may serve as a valuable tool in the clinical decision-making process for patients with CPTs, providing additional prognostic information compared to WHO histologic grade alone. The value of methylation array analysis is particularly important given the lack of consensus on treatment regimens for CPTs.

## Lay Summary

Choroid plexus tumors are rare brain tumors that can be aggressive. Usually, these tumors are diagnosed by examining the tumor under a microscope after surgery. However, this method can sometimes be inaccurate because the appearance of the tumor under the microscope doesn’t always match its aggressiveness. The authors of this study wanted to see if genetic techniques, specifically DNA methylation profiling, could be used to diagnose these tumors and predict their aggressiveness more accurately. To do this, they studied 38 patients with choroid plexus tumors treated at one hospital between 1995 and 2023. They compared traditional microscopic appearance with DNA methylation profiling. Their results showed that DNA methylation profiling generally agreed with the microscopic analysis, but in some cases, it provided additional information that better predicted how aggressive the tumor would be.

**Key Points**

- DNA methylation profiling is a valuable diagnostic tool that can help identify patients with choroid plexus tumors at higher risk of recurrence.
- This study supports the use of DNA methylation profiling in clinical practice as an ancillary test for the diagnosis and management of choroid plexus tumors.

**Importance of Study**

DNA methylation profiling has become an important diagnostic tool in primary CNS tumors. While several studies have shown the prognostic implications of methylation profiling in choroid plexus tumors, the clinical significance of these molecular subgroups in the context of the current therapeutic approaches is unclear. In this study, we evaluated the clinical utility of methylation subgrouping within our pediatric institutional cohort, by incorporating the histopathological, molecular data, and clinical data. We show that DNA methylation profiling can be a valuable ancillary method in predicting the clinical outcomes of these tumors. The integration of conventional histology with the methylation subgroup can improve risk stratification, potentially allowing for identification of patients at high-risk for disease recurrence and augmentation of treatment and/or surveillance strategies.

Choroid plexus tumors (CPTs) are rare intraventricular neoplasms that arise from choroid plexus epithelium and account for <1% of all primary central nervous system (CNS) tumors.^1^ Historically, these tumors were classified based on their histologic features into three distinct entities: choroid plexus papilloma (CPP), atypical choroid plexus papilloma (aCPP), and choroid plexus carcinoma (CPC), with grading conferring significant prognostic and therapeutic implications. Based on the most recent World Health Organization 2021 classification of CNS tumors, CPPs are defined as low-grade papillary lesions with very low or absent mitotic activity, while CPCs are aggressive tumors with frank features of malignancy including sheet-like growth, frequent mitoses, tumor necrosis, and anaplasia.^2^ Atypical CPPs fall somewhere on the spectrum between CPPs and CPCs, typically demonstrating some degree of cellular and architectural atypia, and having two or more mitoses per 10 high-power fields.

Treatment approaches for patients with CPTs have traditionally relied primarily on histologic grade. For all CPTs, surgical intervention is indicated for both diagnostic and therapeutic purposes. Maximal safe resection is the mainstay of treatment, with gross total resection (GTR) potentially curative for CPPs.^3–5^ In contrast, successful treatment of CPCs have typically required a multi-modal approach that include aggressive neoadjuvant chemotherapy and/or radiation therapy.^4–6^ For aCPPs, however, there is a general lack of consensus regarding management strategies, which often results in a significant clinical dilemma, with treatment approaches ranging from observant management without adjuvant treatment to aggressive multi-modal approaches.^7–9^

DNA methylation profiling has emerged in the past decade as an important tool in the diagnostic work-up, molecular subgrouping, and prognostication of CNS tumors.^10^ For CPTs in particular, several recent studies have revealed three distinct methylation subgroups with clinical and prognostic significance.^11,12^ These include “choroid plexus papilloma, pediatric type” (P), “choroid plexus carcinoma, pediatric type” (C), and “choroid plexus tumor, adult type” (A). CPP pediatric type (P) and CPT adult type (A) are associated with a favorable clinical outcome and low risk of disease recurrence. On the contrary, CPC pediatric type (C) portends an unfavorable prognosis with higher risk of recurrence.^11,13^

While DNA methylation profiling-based molecular subgrouping has provided critical insights into the clinical behavior of CPTs, its significance in the context of the current treatment paradigm is not entirely clear. In this study, we sought to evaluate the potential clinical utility of DNA methylation profiling in CPTs by combining histopathological and molecular subgrouping with detailed clinical data, including survival outcomes, within our cohort of patients. This multidisciplinary study aims to refine our understanding of the prognostic significance of these molecular subgroups in the context of current treatment approaches.

## Methods

### Patient Selection and Data Collection

The Institutional Review Board for Boston Children’s Hospital approved this study. Patients diagnosed and treated for CPTs at Dana-Farber Cancer Institute/Boston Children’s Hospital between 1995 and 2023 were identified using our institutional databases. For each case, patient demographics including age at diagnosis, sex, tumor location, resection status (GTR or subtotal resection), disease stage, germline testing (when available), treatment regimen, clinical course, duration of follow-up and survival outcomes were extracted from the medical records.

### Histopathologic Assessment

Two board-certified neuropathologists (SA, CM) reviewed the histology for each case based on the 5th edition of the WHO Classification of the Tumors of the Central Nervous Systems criteria for CPTs. Histologic data including diagnosis, WHO grade, p53 immunohistochemistry results, MIB-1 (Ki-67) proliferation index, available clinical molecular results (next generation sequencing), and DNA methylation profiling were recorded.

### DNA Methylation Profiling

Selected slides (H&E and unstained) were sent to the National Institutes for Health/National Cancer Institute (NIH/NCI) for DNA methylation profiling. Methylation profiling was performed as previously described.^14^ The published DKFZ/Heidelberg classifier^10^ was used to generate tumor classification with a calibrated score.

### Data Analysis

Descriptive statistics were used to summarize patient demographics and clinical characteristics of the patient cohort. Progression-free survival (PFS) was calculated from the date of diagnosis to the date of disease progression. Overall survival (OS) was calculated from the data of diagnosis to the date of death. Patients alive and/or without disease progression were censored at the time of last follow-up. Both PFS and OS were estimated according to the Kaplan–Meier method, and difference in survival were assessed using the log-rank test. Statistical computations were performed using GraphPad Prism v9 (GraphPad Software, San Diego, CA) and the R project (https://www.r-project.org). Riverplot was generated using the RAWGraphs application (https://www.rawgraphs.io).^15^

## Results

**Figure 1.**
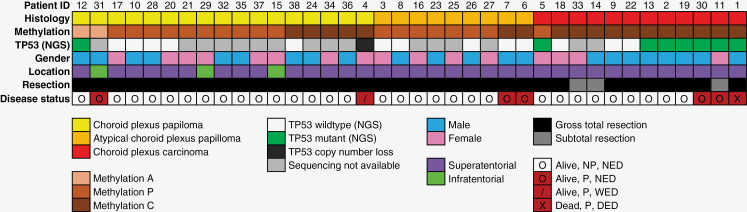
Oncoplot illustrating the cohort with histologic diagnoses, methylation class, and follow-up information. NED—no evidence of disease, WED—with evidence of disease, DED—dead with disease, NP—not progressed, P—progressed, NGS—next-generation sequencing.

A summary of the histologic, molecular, and clinical findings of all patients is illustrated in Table 1, Figure 1, and Supplementary Table 1.

**Table 1.** Summary of the Entire Cohort

Number of patients		Full group	CPP	aCPP	CPC
		38	17 (45%)	9 (24%)	12 (31%)
Patient sex	Male	22 (58%)	9 (53%)	5 (56%)	8 (67%)
	Female	16 (42%)	8 (47%)	4 (44%)	4 (33%)
Age at diagnosis [years; median (range)]		1.5 (0.2–27.7)	3.9 (0.5–27.7)	0.52 (0.2–9.3)	0.75 (0.4–15.5)
Tumor location	Supratentorial (lateral ventricles)	35 (92%)	14 (82%)	9 (100%)	12 (100%)
	Infratentorial (fourth ventricle)	3 (8%)	3 (18%)	0	0
Metastasis	Yes	9 (24%)	2 (12%)	1 (11%)	6 (50%)
	No	29 (76%)	15 (88%)	8 (89%)	6 (50%)
Initial extent of resection	GTR	35 (92%)	17 (100%)	9 (100%)	9 (75%)
	STR	3 (8%)	0	0	3 (25%)
Upfront chemo	Yes	11/37 (30%)	0	0	11/11 (100%)
Upfront RT	Yes	1/37 (2.7%)	0	0	1/11 (9.1%)
Recurrence	Yes	7 (18%)	2 (12%)	2 (22%)	3 (25%)
	No	31 (82%)	15 (88%)	7 (78%)	9 (75%)
Median PFS [months; (range)]		54 (0.8-277)	108 (1.4–222)	36 (8–176)	48 (0.8–277)
Median OS [months; (range)]		79 (0.8–277)	109 (1.4–222)	64 (8–176)	84 (0.8–277)
Median follow-up [months; (range)]		79 (0.8–277)	109 (1.4–222)	64 (8–176)	84 (0.8–277)
Death	Yes	1 (3%)	0	0	1 (8%)
	No	37 (97%)	17 (100%)	9 (100%)	11 (92%)

A total of 48 patients with CPT treated at our institution were identified. Of these, 38 had available tumor material for methylation array and were included in the analysis. The median age at diagnosis of our cohort was 1.5 years (range:0.2–27.7 years). Our cohort was comprised of 17 patients with histologic CPP (45%), nine patients with aCPP (24%), and 12 patients with CPC (31%). Most patients had localized disease; nine patients (24%) presented with metastatic disease. Most patients (92%) underwent initial GTR of their primary disease; three patients with CPC underwent subtotal resections (STRs). Adjuvant treatment data were available for 37 out of 38 patients. One patient with CPC was lost to follow-up early in their treatment course. Eleven patients received adjuvant chemotherapy following initial surgery, all of whom were diagnosed with CPC. The median follow-up period was 79 months [6.6 years; range (0.8–277 months)], the median PFS and OS were 54 months [4.5 years; range (0.8–277 months)] and 79 months [6.6 years; range (0.8–277 months)], respectively. Patient demographic and clinical data are summarized in Table 1, with the treatment summary described in Table 2.

**Table 2.** Histologic Diagnosis, Methylation Class, and Surgical Outcome

Study ID number	Age at diagnosis (years)	Histologic diagnosis	Methylation class	Upfront surgery	Upfront chemotherapy	Upfront radiation therapy	Time to first recurrence (months)
12	27.7	CPP	A	GTR	None	None	
31	9.8	CPP	A	GTR	None	None	24
17	4.0	CPP	P	GTR	None	None	
10	4.5	CPP	P	GTR	None	None	
28	2.1	CPP	P	GTR	None	None	
20	0.5	CPP	P	GTR	None	None	
21	1.7	CPP	P	GTR	None	None	
29	15.6	CPP	P	GTR	None	None	
32	13.9	CPP	P	GTR	None	None	
35	1.0	CPP	P	GTR	None	None	
37	8.3	CPP	P	GTR	None	None	
15	16.7	CPP	P	GTR	None	None	
38	0.6	CPP	C	GTR	None	None	
24	3.9	CPP	C	GTR	None	None	
34	1.1	CPP	C	GTR	None	None	
36	0.7	CPP	C	GTR	None	None	
4	1.8	CPP	C	GTR	None	None	2
3	1.6	aCPP	P	GTR	None	None	
8	1.6	aCPP	P	GTR	None	None	
16	0.4	aCPP	P	GTR	None	None	
23	0.4	aCPP	P	GTR	None	None	
25	0.6	aCPP	P	GTR	None	None	
26	0.3	aCPP	P	GTR	None	None	
27	0.2	aCPP	P	GTR	None	None	
7	0.5	aCPP	C	GTR	None	None	12
6	9.3	aCPP	C	GTR	None	None	11
5	14.6	CPC	P	GTR	SIOP-2009: Arm A	Focal RT 54 Gy	
18	0.5	CPC	C	GTR	SIOP-2009: Arm A	None	
33	15.5	CPC	C	STR	Unknown	Unknown	
14	0.6	CPC	C	STR	PBTC-001 followed by DF 02-294	None	
9	5.8	CPC	C	GTR	HeadStart II with auBMT	None	
22	1.5	CPC	C	GTR	SIOP-2009: Arm A	None	
13	0.5	CPC	C	GTR	SIOP-2000: CycEV	None	
2	0.8	CPC	C	GTR	HeadStart II with auBMT	None	
19	0.7	CPC	C	GTR	PBTC-001 (without RT)	None	
30	0.4	CPC	C	GTR	ACNS0334 ARM A with auBMT	None	19
11	3.8	CPC	C	STR	SIOP-2009: Arm A	None	36
1	0.4	CPC	C	GTR	HeadStart II	None	1

### Choroid Plexus Papilloma

**Figure 2.**
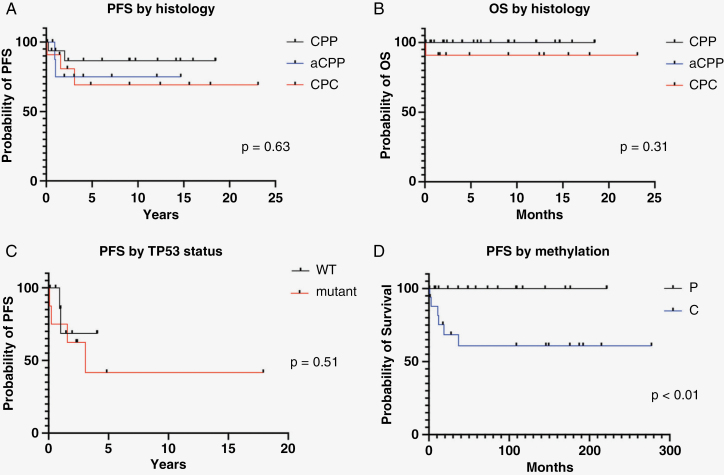
Outcomes of the cohort based on histology (PFS and OS), TP53 status, and methylation class. Note: For Figure 2B, both CPP and aCPP have OS of 100%.

Seventeen cases of CPP were identified. DNA methylation array was performed on all of them. Of these, 10 tumors (59%) matched with CPP pediatric type (P); five tumors (29%) matched with CPC pediatric type (C); and two tumors (12%) matched with CPT adult type (A). All patients had GTR upfront, and none received upfront adjuvant chemotherapy or radiation therapy after surgery. With a median follow-up period of 9.1 years (range:0.1–18.5), the 5- and 10-year PFS were both 86.3%, while the 5- and 10-year OS in this cohort were both 100% (Figure 2A and B).

Two patients experienced disease recurrence in this cohort (Table 2). The first patient (study ID 31) had metastatic disease upfront and underwent GTR of both primary and metastatic lesions. On DNA methylation profiling, this tumor matched to CPT adult type (A), and this patient suffered disease recurrence 2 years after initial presentation. This patient was subsequently salvaged with repeat surgery, followed by adjuvant radiation therapy and chemotherapy, and remains disease-free more than 10 years after recurrence. The second patient (study ID 4) is described in further detail below. Notably, all patients diagnosed histologically with CPP and then sub-grouped on methylation as CPP pediatric type (P) are long-term survivors without disease recurrence.

**Figure 3.**
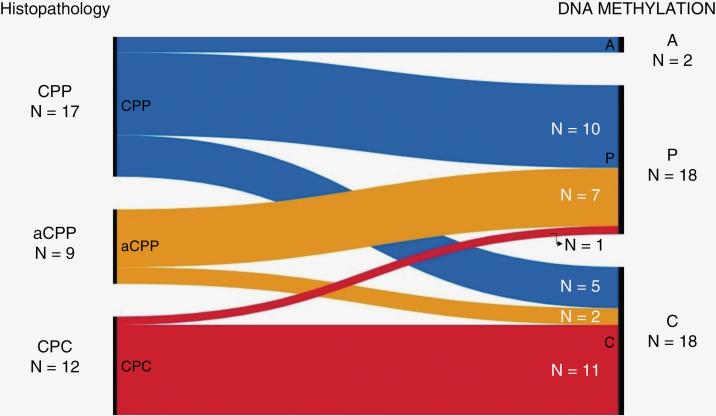
Illustration of the histologic diagnoses (CPP, aCPP, CPC) and corresponding methylation groups (A, P, C) in the cohort.

**Figure 4.**
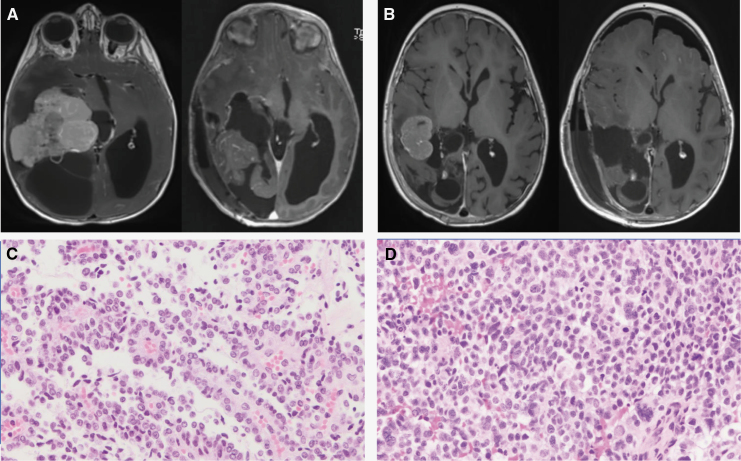
Patient 4. (A) MRI before the first surgery and (B) MRI at recurrence, before the second surgery. (C) Choroid plexus papilloma histology on the first resection specimen, with papillary architecture and very low mitotic rate; (D) Choroid plexus carcinoma histology on the second resection specimen, with loss of papillary architecture, cytologic atypia, and frequent mitoses.

Discrepancy between histology and DNA methylation profiling was seen in six patients with CPP. One pediatric patient (age 9.8) was classified as CPT adult type (A) and suffered disease recurrence as noted above (study ID 31). The other five discrepant cases were classified on methylation as CPC pediatric type (C) (Figure 3). Among these five cases, four are long-term survivors of their disease without evidence of recurrence/progression. The patient with study ID 4 suffered early disease recurrence. At diagnosis, this patient underwent a GTR, and the tumor was diagnosed histologically as a CPP. Although DNA sequencing panel showed a copy loss of *TP53* that was part of a larger region loss on chromosome 17, the reassuring histology and tumor sequencing that lacked *TP53* mutation led to observant management without adjuvant therapy. The DNA methylation profiling result of CPC pediatric type (C) prompted short-interval surveillance scan, which showed significant local recurrence less than two months from initial surgery. The patient underwent repeat GTR, with the histology from the second resection consistent with CPC (Figure 4). Additionally, DNA sequencing of the second resection specimen revealed unequivocal homozygous loss of *TP53*. This patient was salvaged with intensive chemotherapy followed by focal radiation therapy and remains alive with disease more than 2 years after initial diagnosis.

### Atypical Choroid Plexus Papilloma

Of the nine aCPPs, seven matched on methylation with CPP pediatric type (P), and two matched with CPC pediatric type (C). Most patients (8/9, 89%) had localized disease, and all patients underwent GTR (including metastatic sites) without additional adjuvant therapy. With a median follow-up period of 5.3 years (range:0.69–14.67), the 5- and 10-year PFS were 74.8% while the 5- and 10-year OS were 100% (Figure 2A and B).

In this cohort of patients with aCPP, all patients (*n* = 7) who matched with CPP pediatric type (P) were alive at last follow-up without evidence of disease recurrence after initial surgery. Two patients in this cohort experienced disease recurrence within 1 year of diagnosis, and both were classified on methylation testing as CPC pediatric type (C). Both patients were salvaged successfully with chemotherapy ± radiation therapy and remain in remission more than 5 years from initial diagnosis.

### Choroid Plexus Carcinoma

Twelve cases of CPC were identified and sent for DNA methylation array. Of these, 11 matched on methylation to CPC pediatric type (C), while one matched to CPP pediatric type (P). Most patients (9/12, 75%) underwent GTR of their primary disease, while initial staging revealed metastatic disease in half of the cohort. Seven patients in this cohort underwent clinical NGS testing at the time of diagnosis, with six testing positive for *TP53* mutation. Six patients in this cohort tested positive for Li-Fraumeni Syndrome (LFS) on germline testing. Of note, this includes one patient who did not undergo somatic NGS testing and subsequently tested positive for germline *TP53.*

All 11 CPC patients with follow-up data received adjuvant chemotherapy. One patient received focal radiotherapy after completion of adjuvant chemotherapy (Table 2). With a median follow-up period of seven years (range:0.1–23.1), the 5- and 10- year PFS were 69%, while the 5- and 10-year OS were 90.7% (Figure 2A and B). Three patients had disease recurrence, all of whom matched to CPC pediatric type (C). None had received radiotherapy upfront. All three had *TP53* mutation on NGS and two were subsequently confirmed to have germline Li Fraumeni Syndrome. One patient died soon after disease progression, while the other two were successfully salvaged with radiation therapy and chemotherapy.

Discrepancy between histology and DNA methylation profiling was seen in one case, where one patient with CPC matched to CPP pediatric type (P). This patient (Study ID 5) received upfront chemotherapy followed by focal radiation therapy and remains in remission almost 5 years since initial diagnosis. Of note, this patient had *TP53* mutation on NGS and was subsequently found to have Li Fraumeni Syndrome.

### Survival Outcomes Based on Molecular Features

Seventeen cases underwent DNA-based next generation sequencing. *TP53* mutations/deletions were identified in eight cases (histologically 6 CPC, 2 CPP; methylation group 6 C, 1 A, and 1 P). *TP53* mutations were not identified in the other nine cases, which included 4 CPPs, 4 aCPPs, and 1 CPC (5 P and 4 C on methylation). There was no statistically significant difference in survival for the patients with *TP53* mutations when compared with those without. (*P* = .51) (Figure 2C).

There was a significant difference in survival between patients with tumors that matched with methylation class CPP pediatric type (P) versus those that matched to CPC pediatric type (C) (*P* = .02) (Figure 2D).

## Discussion

The advancement of cancer genomics has significantly furthered our understanding of the molecular underpinnings of various malignancies, allowing for better prognostication, and improved treatment. DNA methylation profiling has emerged as a reliable methodology to diagnose primary CNS tumors, and refined our ability to distinguish molecular subgroups.^10^ Recent studies have demonstrated the potential for DNA methylation profiling in the molecular subgrouping of CPTs, with reported clinical and prognostic significance.^11,12,16^ In this study, we aimed to improve this understanding by analyzing and correlating the outcomes of patients treated with conventional approaches with the DNA methylation-based molecular subgroups.

Our data shows that in the majority of cases, the DNA methylation subgroups correlated well with conventional histopathology, with most cases of histologically defined CPP and CPC matching their respective methylation class. In addition, despite discrepancies identified in several cases across all three histological types, the survival outcomes seen in all three histological subtypes are similar to those previously published in the literature.^3,5,17–20^ DNA methylation profiling was particularly effective in predicting the outcome in the aCPP group, which was uniformly treated with surgery alone and observation upfront. In this cohort, only the two cases that matched to CPC pediatric type (C) had disease recurrence, while those that matched to CPP pediatric type (P) remained disease free. This finding is particularly important for patients with aCPP, as there is a lack of a universally accepted standard-of-care approach for these patients. While there are institutions that have reported reasonable outcomes with conservative treatment approaches and expectant observant management, others have advocated for a proactive multi-modal aggressive approach for a subset of patients.^7–9,21^ Our cohort of patients with aCPP were uniquely positioned for this analysis, as they were all treated in a uniform fashion with GTR only and no adjuvant therapy. Our data supports a more conservative approach for patient with aCPP that matched to methylation class CPP pediatric type (P), and suggests that patients with aCPP, methylation class CPC pediatric type (C) may be at higher risk for early disease recurrence and could benefit from a more aggressive surveillance approach.

Despite the discrepancies noted between histology and methylation class among CPPs, the overall outcome of this cohort of patient remains excellent. This includes four cases that were classified on methylation as CPC pediatric type (C), further highlighting the value and prognostic significance of histopathology. That said, the case highlighted above (study ID 4) is particularly noteworthy. Shortly after initial GTR and observant management for a histologically defined CPP, this patient had a local recurrence with histologic and molecular progression to CPC. This case illustrates a rare occurrence of malignant evolution/transformation of CPP, which has been reported in several studies.^22–24^ Importantly, DNA methylation testing of the initial CPP tumor matched the tumor to CPC pediatric type (C), which substantiates the ability of methylation profiling to identify patients who are at a higher risk for disease recurrence. In the case, the results of DNA methylation profiling led to augmentation of the surveillance strategy that we adopted for this patient, which allowed for earlier detection of disease recurrence and a timely change in the treatment approach.

Our study has several limitations, which are predominantly related to the rarity of this disease and retrospective study design. The retrospective nature of the study increases the risk of selection and information bias and decreases the uniformity of treatment approaches (ie, chemotherapy regimen) among different patients. The rarity of the disease means that our sample size is relatively small, which limits the ability to reach definitive conclusions. Nonetheless, our study provides critical insight into the potential utility of DNA methylation subgrouping for CPTs, as well as its implications on treatment approach, prognostication, and surveillance strategies.

## Conclusion

Our study demonstrates that DNA methylation profiling is a valuable ancillary method in the diagnosis of CPTs, correlating well with the histologic diagnosis and is predictive of clinical outcome. It is potentially most impactful in choroid plexus tumors with atypical histology, where it could be useful in risk stratification and clinical management. For patients with CPP, DNA methylation profiling may help inform the risk for disease recurrence, and potentially support the augmentation surveillance strategies.

## Supplementary Material

vdae097_suppl_Supplementary_Materials

## Data Availability

Data from this study will be made available upon reasonable request.
