# Supplementary material for: Clinical utility of DNA methylation profiling for choroid plexus tumors
Source: Neurooncol Adv. 2024 Jun 12;6(1):vdae097. doi: 10.1093/noajnl/vdae097 (PMC11221062; doi:10.1093/noajnl/vdae097)
Supplement: vdae097_suppl_Supplementary_Materials [file vdae097_suppl_Supplementary_Materials.docx]

| Study ID | Histologic diagnosis | Methylation group | Mitotic index | Proliferation index (Ki67) |
| --- | --- | --- | --- | --- |
| 17 | CPP | Ped A(P) | 1 | 3 |
| 31 | CPP | Adult (A) | 0 | 2 |
| 12 | CPP | Adult (A) | 1 | 2 |
| 10 | CPP | Ped A(P) | 1 | 1 |
| 28 | CPP | Ped A(P) | 0 | 1 |
| 20 | CPP | Ped A(P) | 0 | 3 |
| 21 | CPP | Ped A(P) | 0 | 2 |
| 29 | CPP | Ped A(P) | 0 | 1 |
| 32 | CPP | Ped A(P) | 0 | 1 |
| 35 | CPP | Ped A(P) | 1 | 3 |
| 37 | CPP | Ped A(P) | 0 | 2 |
| 15 | CPP | Ped A(P) | 0 | 1 |
| 4 | CPP | Ped B(C) | 1 | 5 |
| 38 | CPP | Ped B(C) | 1 | 5 |
| 24 | CPP | Ped B(C) | 0 | 2 |
| 34 | CPP | Ped B(C) | 1 | 3 |
| 36 | CPP | Ped B(C) | 0 | 1 |
| 5 | CPC | Ped A(P) | 6 | 5 |
| 30 | CPC | Ped B(C) | 20 | 30 |
| 18 | CPC | Ped B(C) | 17 | 40 |
| 33 | CPC | Ped B(C) | 12 | 20 |
| 14 | CPC | Ped B(C) | 13 | 22 |
| 9 | CPC | Ped B(C) | 14 | 30 |
| 22 | CPC | Ped B(C) | 15 | 20 |
| 13 | CPC | Ped B(C) | 18 | 30 |
| 2 | CPC | Ped B(C) | 14 | 40 |
| 1 | CPC | Ped B(C) | 32 | 35 |
| 11 | CPC | Ped B(C) | 35 | 88 |
| 19 | CPC | Ped B(C) | 8 | 25 |
| 3 | aCPP | Ped A(P) | 2 | 7 |
| 8 | aCPP | Ped A(P) | 7 | 17 |
| 16 | aCPP | Ped A(P) | 6 | 12 |
| 23 | aCPP | Ped A(P) | 12 | 35 |
| 25 | aCPP | Ped A(P) | 3 | 9 |
| 26 | aCPP | Ped A(P) | 2 | 2 |
| 27 | aCPP | Ped A(P) | 10 | 20 |
| 7 | aCPP | Ped B(C) | 3 | 15 |
| 6 | aCPP | Ped B(C) | 6 | 30 |
